# Pulmonary thromboembolism associated with hereditary antithrombin III deficiency: A case report

**DOI:** 10.1097/MD.0000000000037429

**Published:** 2024-03-08

**Authors:** Jingwei Liu, Yin Wang, Chunyan Rong, Baoguo Wang, Xuhan Liu, Weihua Zhang

**Affiliations:** a Department of Cardiovascular Medicine, The First Hospital of Jilin University, Changchun, China.

**Keywords:** anticoagulation, antithrombin deficiency, hereditary thrombophilia, pulmonary embolism, *SERPINC1*

## Abstract

**Background:**

Thrombophilia is a coagulation disorder closely associated with venous thromboembolism. Hereditary antithrombin III (AT III) deficiency is a type of genetic thrombophilia. In China, genetic thrombophilia patients mainly suffer from deficiencies in AT III, protein S, and protein C. Multiple mutations in the serpin family C member 1 (*SERPINC1*) can affect AT III activity, resulting in thrombosis.

**Case presentation:**

This case presented a 17-year-old adolescent female who developed lower extremity venous thrombosis and subsequently pulmonary embolism (PE) following a right leg injury. A missense mutation in gene *SERPINC1* of c.331 T > C, p.S111P was detected on the patient, resulting in a decreased AT III activity and an elevated risk of thrombosis. The patient received anticoagulation treatment for approximately 5 months. During follow-up, the blood clot gradually dissolved, and there have been no recurrent thrombotic events reported thus far.

**Discussion:**

Hereditary AT deficiency can be classified into two types based on the plasma levels of the enzymatic activity and antigen. Type I is a quantitative defect, while Type II is a qualitive defect. Until 2021, 486 *SERPINC1* gene mutations have been registered, more than 18% of which are point mutations. The *SERPINC1* mutation c.331 T > C in was firstly reported in 2017, which was classified into type I AT III deficiency.

**Conclusion:**

Hereditary thrombophilia is a coagulation disorder with a high omission diagnostic rate. Minor mutations in the *SERPINC1* gene can also lead to hereditary AT III deficiency, which in turn can cause PE. We emphasized the importance of etiological screening for hereditary thrombophilia in venous thromboembolism patients without obvious high-risk factors. Long-term anticoagulation treatment and avoidance of potential thrombosis risk factors are critical for such patients.

## 1. Introduction

Thrombophilia refers to a coagulation disorder characterized by an increased tendency of blood clotting. The disorder is closely associated with an increased risk of venous thromboembolism (VTE), including deep venous thrombosis (DVT) and pulmonary embolism (PE), which affects approximately 0.1% to 0.2% of people per year. Thrombophilia is influenced by both inherited factors and acquired factors. Common acquired risk factors of thrombophilia include antiphospholipid antibody syndrome, neoplasia, oral contraceptive use, obesity, smoking and surgery.^[1]^

Hereditary thrombophilia is prevalent in approximately 7% of the general population.^[2]^ It is usually associated with gene mutations in physiological anticoagulant protein function deficiency, such as antithrombin (AT), protein C (PC) and protein S (PS), or gene mutations in procoagulant proteins, such as factor V Leiden (FVL) mutation and the prothrombin gene mutation G20210A.^[3]^ FVL mutation is the most common genetic risk factor for VTE, which is found in 50% of patients with familial thrombophilia.^[4]^ In China, genetic causes of thrombophilia mainly include deficiencies in AT, PC, and PS.^[3]^

Antithrombin deficiency is a rare hereditary disease caused by mutations of AT gene (*SERPINC1*), which is located on chromosome 1q23 to 25 and spans 13.5 kb in the genome. The mutation can lead to reduced content or decreased activity of AT and finally contributes to thrombosis.^[5]^ We report a case of PE caused by the decreased activity of AT due to a mutation in *SERPINC1* encoding antithrombin III.

## 2. Case report

A 17-year-old female patient was admitted to the Department of Cardiovascular Medicine of the First Hospital of Jilin University on September 30, 2022, with complaints of pain and swelling in the right knee for half a month after falling, accompanied by dyspnea for 5 days. The patient fell down while riding an electric bicycle half a month ago and injured her right knee. Her right knee was swelling and painful but did not affect daily activities. These symptoms worsened 5 days ago accompanied by dyspnea. She has no significant past medical history.

Physical examination on admission revealed the body temperature was 36.5°C, the pulse rate was 112 beats per minute, the breathing rate was 20 beats per minute, blood pressure was 107/70 mm Hg (1 mm Hg = 0.133 kPa). She has no jugular venous distention. There were coarse breath sounds in both lungs, but no crackles were heard. Her heart rhythm was regular, and no additional heart sounds and cardiac murmurs were heard. There was no edema in both lower extremities. The right knee was swelling.

Lower extremity vascular ultrasound revealed acute-staged venous thrombosis in superficial femoral vein, popliteal vein and intermuscular vein of right lower extremity. Computed tomography pulmonary angiography (CTPA) indicated the embolism in the middle branch of right pulmonary artery and lower branch of double pulmonary artery. Computed tomography angiography revealed no atrial and ventricular enlargement.

Laboratory findings are shown in Table 1. D-dimer and fibrinogen (FGB) were significantly increased, whereas cardiac troponin I (cTnI) and B type natriuretic peptide (BNP) was in the normal range. Blood gas analysis suggested a respiratory alkalosis with pH of 7.47 and PCO_2_ of 34 mm Hg. The patient’s coagulation function, platelet (PLT) amount, PS and PC activity, and antibodies about connective tissue diseases and vasculitis are all normal.

**Table 1:** The significant laboratory test results at the first admission.

Parameter	Values	Reference range	Unit
cTnI	<0.05	0–0.05	ng/mL
Myoglobin	60.0	0–107	ng/mL
BNP	15.5	0–100	pg/mL
FBG	5.23	1.8–4.0	g/L
D-dimer	4550	100–600	ng/mL
PH	7.47	7.35–7.45	
PCO_2_	34	35–48	mm Hg
PO_2_	92	83–108	mm Hg
SO_2_C	98	93–98	%
WBC	8.82	3.50–9.50	10^9^/L
MO%	0.07	0.03–0.10	%
EO%	0.10	0.004–0.08	%
PLT	252	125–350	10^9^/L
HB	122	115–150	g/L
Thrombin time (TT)	14.9	11.0–21.0	s
Prothrombin time (PT)	11.0	9.0–13.0	s
Activated partial thromboplastin time (APTT)	25.8	21–33	s
International normalized ratio (INR)	0.95	0.8–1.2	
Prothrombin activity (PTA)	110	80–120	%
Albumin	40.4	42.0–56.0	g/L
Alanine transaminase (ALT)	25.3	10.0–31.0	U/L
Creatinine	54.1	39–76	µmol/L
C-reactive protein (CRP)	109.74	0–1.0	mg/L
Rheumatoid factor (RF)	10.36	0–14.00	IU/mL
IgG antibody	11.22	8.6–17.4	g/L
IgA antibody	1.60	1.0–4.2	g/L
IgM antibody	1.40	0.5–2.8	g/L
Complements C3	1.9	0.7–1.4	g/L
Complements C4	0.51	0.1–0.4	g/L
Anticardiolipidn IgG antibody	2	0–12	GPL
Anticardiolipidn IgA antibody	1	0–12	APL
Anticardiolipidn IgM antibody	10	0–12	MPL
Perinuclear anti-neutrophil cytoplasmic antibody (pANCA)	<1:10		
Cytoplasmic anti-neutrophil cytoplasmic antibody (cANCA)	<1:10		
Myeloperoxidase antibody (Anti-MPO)	2.39	0–20	Units
Proteinase 3 antibody (Anti-PR3)	3.50	0.00–20.00	Units
Double-stranded DNA antibody (anti-dsDNA)	Negative	Negative	
AT III activity	43.5	80.0–130.0	%
PS activity	133.3	63.5–149.0	%
PC activity	93	70–140	%

ALT = alanine transaminase, Anti-dsDNA = double-stranded DNA antibody, Anti-MPO = myeloperoxidase antibody, Anti-PR3 = proteinase 3 antibody, APTT = activated partial thromboplastin time, BNP = B type natriuretic peptide, cANCA = cytoplasmic anti-neutrophil cytoplasmic antibody, CRP = C-reactive protein, cTnI = cardiac troponin I, FBG = fasting plasma glucose, HB = haemoglobin, INR = international normalized ratio, pANCA = perinuclear anti-neutrophil cytoplasmic antibody, PLT = Platelet, PT = prothrombin time, PTA = prothrombin activity, RF = rheumatoid factor, TT = thrombin time, WBC = white blood cell.

The AT III activity was detected in SYSMEX coagulation analyzer CS-5100-5 with the substrate luminescence method. The patient’s peripheral blood 2 mL was mixed with 0.11 mol/L sodium citrate solution at 9:1, and centrifugation for 15 minutes at a rate of 3000 rpm. The plasma 10 µL was isolated and mixed with the thrombin reagent 600 µL and incubated at 37°C for 3 minutes. Add the color developer 100 µL, and the change of absorbance was detected at 405 nm. The AT III activity was measured at 43.5% (reference range 80–130%).

We then detected the AT III activity of the patient’s mother, a 39-year-old woman. She never felt chest pain or dyspnea and had no history of DVT or PE. The PS and PC activity was normal, and the AT III activity was measured as 43.9%.

**Figure 1.**
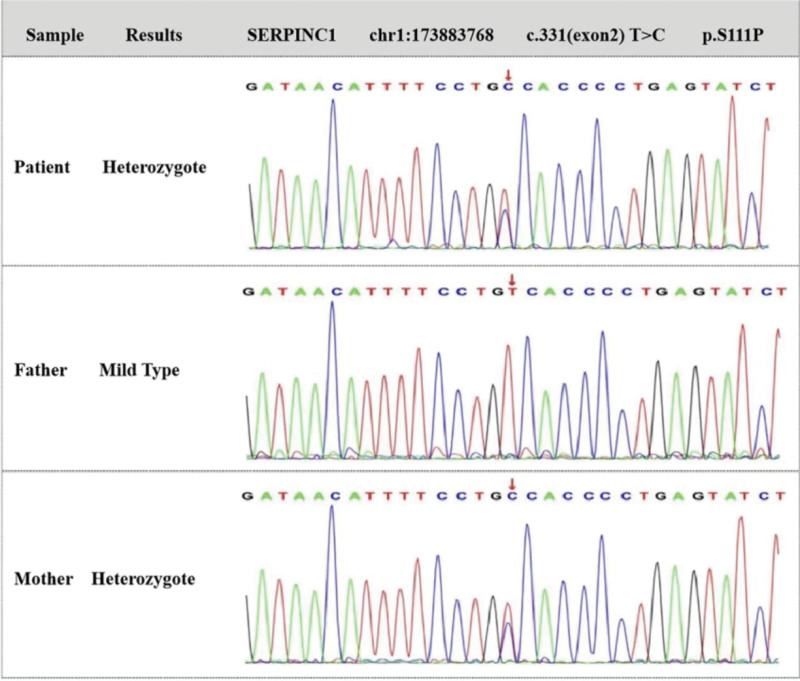
Full exon DNA sequencing results. Due to Sanger’s verification using forward sequencing or reverse sequencing, the base shown in the peak graph may be the reverse complementary sequence of the detected base. The patient and her mother were heterozygote with *SERPINC1* (NM_000488) gene mutations of c.331(exon2) T > C, p.S111P (p.Ser111Pro). The thymine at the 331st position in the *SERPINC1* sequence was replaced by cytosine, resulting in the serine at the 111th position of the amino acid sequence encoded by *SERPINC1* changing to proline. *SERPINC1* = serpin family C member 1.

The patient is a young woman with no special past history. Her parents are not married to close relatives. There was no obvious risk factor of thrombosis besides the decreased AT III activity. Considering a high possibility of hereditary AT III deficiency, full exon deoxyribonucleic acid (DNA) sequencing was further conducted for the patient and her parents (Table 2). In the PCR experiment, the reaction system includes 10 µM F Primer 1.25 µL, DNA polymerase 0.1 µL, 5 X bµffer A 5 µL, 10 mM dNTP 0.5 µL, 10 µM R Primer 1.2 5 µL, DNA 1 µL, and PCR-grade water 15.9 µL. The reaction conditions are as follows: pre-degeneration at 95°C for 3 minutes, degeneration at 95°C for 30 seconds, annealing at 60°C for 30 seconds, and extension at 72°C for 30 seconds. A total of 30 cycles were carried out, then the reaction system was extended at 72°C for 1 minute and preserved at 4°C. The *SERPINC1* (NM_000488) gene single heterozygous mutations of c.331(exon2) T > C, p.S111P (p.Ser111Pro) was detected in both the proband and her mother, while her father was a wild type (Fig. 1).

**Table 2:** Primer sequence of *SERPINC1* (NM_000488) gene in full exon DNA sequencing.

Target site	Primer sequence (5′→3′)	Amplify fragment length (bp)	Annealing temperature (°C)
Exon2	F: CAGTGTTGGTTGAGGAATCATTGG R: GAGAAGAAGGCAACTGAGGATGAG	326	60

DNA = deoxyribonucleic acid, *SERPINC1 =* serpin family C member 1.

**Figure 2.**
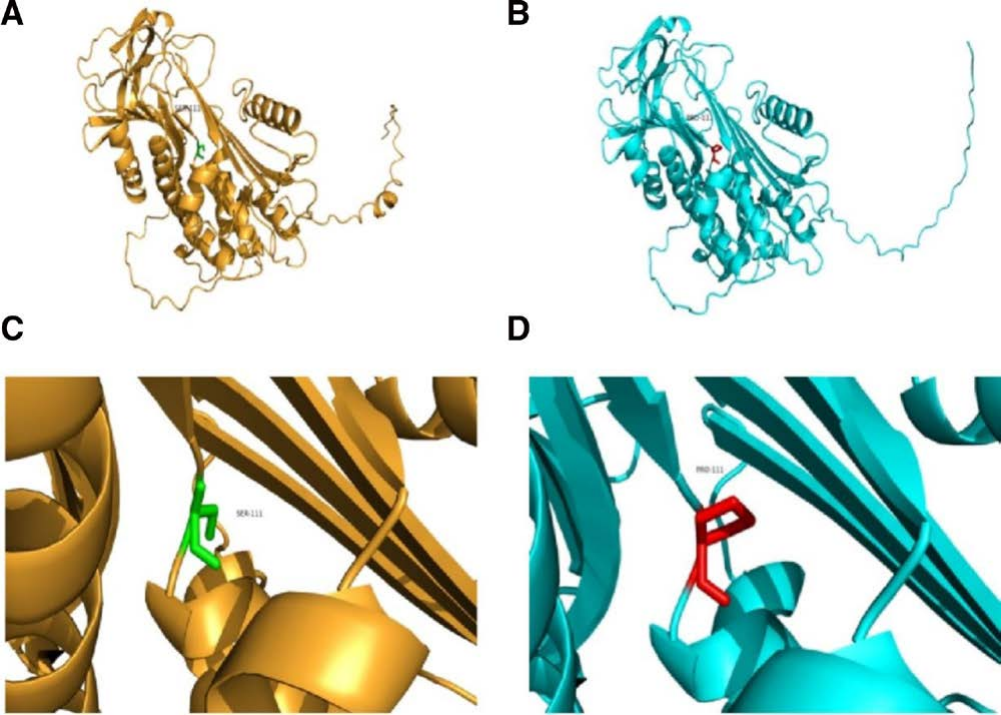
Comparison of 3D structural homology models of wild type and mutant type of AT III. (A, C) Wild type of AT III. The 111th amino acid was serine. (B, D) mutant type of AT III. The 111th amino acid changed from serine to proline. AT = antithrombin.

We forecast the mutated gene and protein function through Mutation Taster (http://www.mutationtaster.org), PolyPhen-2 and REVEL software. The results indicated *SERPINC1* mutation c.331 T > C was harmful. Among them, the pathogenicity rate predicted by Mutation Taster is about 1.000. The closer the probability is to 1, the greater the disease may be, and the more credible the prediction results are. Through Swiss-Model software, the tertiary structure of AT III wild type and mutant type were modeled. The 111th amino acid in the protein encoded by *SERPINC1* was changed from serine to proline (Fig. 2).

The diagnosis of this patient was pulmonary embolism (low-risk group). After admission, the patient was instructed to raise the right lower limb and received the oxygen inhalation (through a nasal catheter, 3–4 L/min). Initially, the patient was administered a subcutaneous injection of enoxaparin sodium, 40 mg twice a day. However, due to the patient’s deficiency in AT III, the anticoagulant treatment was changed to a subcutaneous injection of Fondaparinux Sodium, 2.5 mg once a day. After discharge, the patient’s treatment was transitioned to oral rivaroxaban, initially at a dose of 15 mg twice a day, which was adjusted to 20 mg once a day 3 weeks later.

We followed up with the patient until February 2023. In December 2022, the lower extremity blood vessel color ultrasound revealed partial recanalization after deep vein thrombosis in the right lower extremity. CTPA results indicated no significant abnormalities in pulmonary artery. In February 2023, the patient’s AT III activity was measured at 48%. The patient remained asymptomatic for dyspnea, and there were no limitations in her daily activities.

## 3. Discussion

Inherited thrombophilia is a genetic tendency of VTE. The incidence of hereditary thrombophilia is lower than that of acquired thrombophilia. The most common causes of genetic thrombophilia are the FVL and the prothrombin gene mutation G20210A, accounting for about 50% to 70% of the diagnosed cases.^[6]^ However, there are significant ethnic differences in hereditary thrombophilia. Mutations of FVL and prothrombin gene G20210A are more common in White Caucasians, while mutations in natural anticoagulants are more common in Asians.^[7]^ According to the investigation and research on hereditary thrombophilia in China, the main cause of thrombophilia is anticoagulation protein defects, especially the mutations of *SERPINC1, PROC*, and *PROS1*. Non-type O blood is considered as the most common genetic risk factor associated with VTE,^[6]^ because type O blood group individuals have lower von Willebrand factor and factor VIII levels than non-type O blood group subjects. The prevalence of non-type O blood individuals were approximately twice higher in VTE than controls.^[8]^

This case presented a 17-year-old adolescent female who developed lower extremity venous thrombosis and subsequently pulmonary embolism following a right leg injury. The type B blood makes it is more likely to develop VTE for her. Her antithrombin III activity was found to be decreased significantly, and further genetic testing indicated a heterozygous missense mutation in *SERPINC1*: c.331T > C (p.S111P).

AT is a serine protease inhibitor (serpin) which inhibits multiple coagulation serine proteases, such as activated factor X (FXa) and thrombin (FIIa).^[9]^ AT has 2 major functional domains: the reactive site (RS) responsible for the coagulation factor inhibition, and the heparin-binding site (HBS). The inhibitory function of AT can be enhanced at least 1000-folds when exogenous heparin or heparin sulfate binds to HBS.^[10]^ AT III is a plasmatic α-glicoprotein formed by a single peptidic chain. As the main component of AT, AT III inhibits FIIa and free Xa, IXa, VIIa plasmatic factors.^[11]^ The mass concentration of AT III is 112 to 140 μg/mL, and the reference range of AT III activity is 80% to 120%. The value below 80% means that the AT III activity decreases.^[12]^ It is reported that the prevalence of AT deficiency in the total population is 1/5000 to 1/500.^[13]^ Patients with AT deficiency have a high risk for a first occurrence and recurrence of VTE.^[10]^ AT deficiency is responsible for congenital gene mutations or acquired factors in clinical scenarios causing low hepatic production, protein consumption or protein loss.^[9]^ Hereditary AT deficiency is an autosomal dominant disease, most of them are heterozygotes. The homozygotic of AT deficiency is rare because it is almost fatal in the uterus.^[14]^ Hereditary AT deficiency can be classified into two types based on the plasma levels of the enzymatic activity and antigen.^[10]^ Type I is a quantitative defect characterized by a reduced plasma AT III antigen levels and AT III activity. Type II is a qualitive defect characterized by AT III structural changes, reduced AT III activity, and normal antigen levels, which is divided into three subtypes: RS variations, HBS variations, and multiple variations.^[14,15]^ Studies have reported that the incidence of type I is significantly lower than type II. The risk of thrombosis in type I patients is four times higher than that of type II. Therefore, we should pay more attention to type I patients.^[12]^ Due to the limited detection technology, AT III antigen could not be detected, so it is hard to determine the classification of this patient.

*SERPINC1* mutation is an important factor leading to hereditary AT III deficiency, whose incidence in general population is 0.2‰ to 0.5‰, and in VTE patients is 1% to 8%.^[12]^ Until April 2021, 486 *SERPINC1* gene mutations have been registered in HGMD Professional Edition (https://www.hgmd.cf.ac.uk/ac/gene.php?gene=SERPINC1)mainly including missense mutations, nonsense mutations, splice site mutations, insertion, frameshift mutations, etc. More than 18% of these mutations are point mutations. Relevant studies have shown that even small mutations in *SERPINC1* can cause hereditary AT III deficiency, and almost all deletions and nonsense mutations lead to hereditary AT III deficiency of type I.^[16–19]^ The *SERPINC1* mutation c.331 T > C in this patient was firstly reported in 2017, which was classified into type I AT III deficiency with AT III activity of 49% and mean AT antigen of 52%.^[5]^ We further conducted gene mutation and protein function prediction whose results revealed the mutation was harmful.

This report presents a case of pulmonary embolism caused by a mutation in the *SERPINC1* gene, resulting in decreased AT III activity. Based on this case, for younger patients without obvious high-risk factors for pulmonary embolism, it is recommended to conduct initial phenotypic testing for hereditary thrombophilia, such as AT III, PS, and PC activity. Current methods for AT activity detection are based on chromogenic substrates and evaluate anti-FXa or anti-FIIa anticoagulant activity of plasma AT.^[9]^ If a decrease is observed and not explained by an acquired etiology, the quantification of AT antigen will help to distinguish the quantitative or qualitative AT deficiency. But the results are influenced by acute phase of thrombosis or taking direct oral anticoagulants. It is recommended to realize thrombophilia testing between the third and sixth months after a VTE event. The AT deficiency tests are appropriate for symptomatic patients with clinical suspicion of a major thrombophilia to guide antithrombotic management and avoid recurrence, and for asymptomatic family members of a proband with known congenital antithrombin deficiency to prevent the risk factors of thrombosis and avoid the first event.^[10]^ If abnormalities are detected, genetic testing should be further conducted.

During the acute phase of VTE, antithrombin supplementation can be used besides heparin. After the acute period, oral anticoagulation may be used for long-term anticoagulation treatment, which aims to prevent thrombosis recurrence and secondary thrombotic episodes. The duration of anticoagulation therapy for hereditary antithrombin deficiency is mostly 3 to 6 months, while in some cases, lifelong anticoagulation is required.^[1]^ All AT deficiency carriers should be educated to avoid potential reversible risk factors of thrombosis in their daily life, such as hormonal therapies. Apart from that, individuals with no personal history of thromboembolism or those with a positive history but not taking long-term oral anticoagulation, should receive prophylaxis with heparin and/or antithrombin supplementation at high thrombogenic situations, such as immobilization, surgery, pregnancy and post-partum.^[9]^

In this study, we diagnosed a young female patient with PE caused by hereditary AT III deficiency through phenotypic testing, genetic testing, and protein function prediction. We detected a rare point mutation in gene *SERPINC1* encoding AT III, resulting in a decreased AT III activity and an elevated risk of thrombosis. The same point mutation was also detected on the patient’s mother. The patient received anticoagulation treatment for approximately 5 months. During follow-up, the blood clot gradually dissolved, and there have been no recurrent thrombotic events reported thus far.

The study’s limitation lies in our inability to detect the AT III antigen content and to further categorize the AT III deficiency in this patient. Given the elevated thrombosis risk in type I patients, additional researches are necessary to ascertain whether the duration of anticoagulation therapy should be prolonged relatively for this subgroup. Moreover, in patients with hereditary AT III deficiency patients who has a history of thrombosis, the necessity of lifelong anticoagulant therapy needs further exploration.

## 4. Conclusion

Hereditary thrombophilia is a coagulation disorder with a high omission diagnostic rate. Minor mutations in the *SERPINC1* gene can also lead to hereditary AT III deficiency, which in turn can cause PE. We emphasized the importance of etiological screening for hereditary thrombophilia in VTE patients without obvious high-risk factors. Long-term anticoagulation treatment and avoidance of potential thrombosis risk factors are critical for such patients.

## Acknowledgments

We are most grateful to all participants in the present study.

## Author contributions

**Conceptualization:** Weihua Zhang.

**Formal analysis:** Yin Wang.

**Methodology:** Yin Wang, Chunyan Rong.

**Software:** Baoguo Wang.

**Supervision:** Chunyan Rong, Xuhan Liu, Weihua Zhang.

**Visualization:** Baoguo Wang.

**Writing – original draft:** Jingwei Liu.

**Writing – review & editing:** Xuhan Liu, Weihua Zhang.
